# TRIM proteins: A ‘swiss army knife’ of antiviral immunity

**DOI:** 10.1371/journal.ppat.1013147

**Published:** 2025-05-12

**Authors:** Elise Chabot, David Durantel, Julie Lucifora

**Affiliations:** 1 CIRI, Centre International de Recherche en Infectiologie, Univ Lyon, Inserm, U1111, Université Claude Bernard Lyon 1, CNRS, UMR5308, ENS de Lyon, Lyon, France; 2 Master de Biologie, École Normale Supérieure de Lyon, Lyon Cedex, France; NYU Grossman School of Medicine: New York University School of Medicine, UNITED STATES OF AMERICA

## Abstract

With their modular structure and E3 ubiquitin ligase activity, Tripartite motif (TRIM) proteins interact with a wide range of cellular and viral substrates. This review summarizes how they have emerged as key players in the antiviral response. Shortly, TRIM proteins were shown (i) to enhance pro-inflammatory cytokines production by interacting with pattern recognition receptors and downstream components of immune signaling pathways, (ii) to interfere with viral trafficking by interacting with the cytoskeleton, and (iii) to exhibit direct antiviral effects by targeting viral proteins for proteasomal degradation or inducing autophagy. This combination of actions underscores TRIMs as a potent innate defense system, but also makes them vulnerable to viral evasion strategies.

## 1. Introduction

Viruses are highly diverse infectious agents that target all forms of life and can cause severe diseases. Over time, coevolution between vertebrates and viruses has led to the selection of varied, sophisticated and redundant cellular defenses against these pathogens. Among these defenses, the tripartite motif (TRIM) protein family has been the subject of intense research in the past few years. While *TRIM*-like genes are found in invertebrates such as *Drosophila*, *C. elegans*, and Cnidarians, the TRIM family underwent a significant diversification in vertebrates [1]. This diversification, coinciding with the evolution of the interferon (IFN) pathway and the adaptive immune system, has resulted in a large protein family, central to host-pathogen interactions.

**Fig 1:**
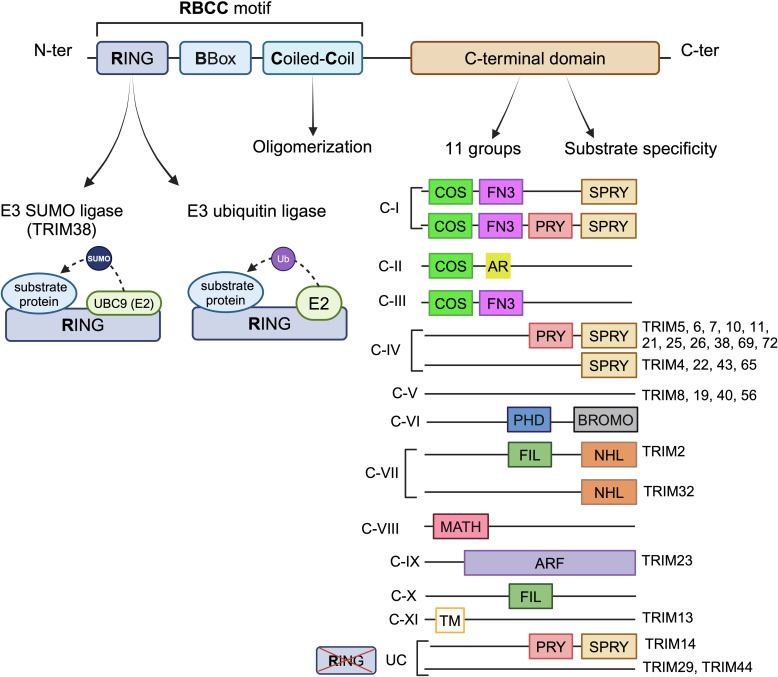
Structure of TRIM proteins. The general structure of TRIM proteins is represented at the top of the figure, with the functions associated to each domain. Only the TRIM proteins mentioned in this review are listed in this figure. Ub: ubiquitin; SUMO: Small Ubiquitin-like Modifier; COS: C-terminal subgroup One Signature; FN3: fibronectin type III domain; AR: acid-rich region; SPRY: SPIa and the ryanodine receptor domain; PRY: SPRY-associated domain; PHD: plant homeodomain; BROMO: bromodomain; FIL: filamin-type IG domain; NHL: NHL repeats; MATH: meprin and tumor necrosis factor receptor–associated factor homology domain; ARF: ADP-ribosylation factor family domain; TM: transmembrane region; UC: unclassified. Created in BioRender. Lucifora, J. (2025) https://BioRender.com/j55c338.

The TRIM protein family is a large group of proteins encoded by more than 80 human genes, some of these genes producing multiple isoforms [2]. TRIM proteins are involved in a variety of cellular processes including cell cycle regulation, transcription and most notably, innate immune response against viral infections. These proteins share a conserved structure at their N-terminal extremity, composed of 3 domains, known as **R**ING-**B**Box-**c**oiled **c**oil (RBCC) motif (Fig 1) [3]. The RING domain confers the E3 ubiquitin ligase activity. Nevertheless, some TRIM proteins, such as TRIM14, TRIM29 and TRIM44, lack this domain [4]. TRIM proteins possess one or two BBox domains whose function remains unclear and a coiled-coil domain that mediates oligomerization, which is often essential for their activity [3]. The C-terminal extremity is highly variable among this family and confers substrate specificity, leading to a classification in 11 groups, from Class I to Class XI [5]. The most common C-terminal domain is PRY-SPRY, present in around 40 TRIM proteins. Notably, the C-terminal domain of TRIM23 contains an ADP-ribosylation factor (ARF) domain, conferring a GTPase activity [6]. Most TRIM proteins exert E3 ubiquitin ligase activity, which consists in the transfer of ubiquitin from a donor E2 ubiquitin-conjugating enzyme to a lysine residue of the targeted protein [7]. Ubiquitin contains several lysine residues, enabling the formation of polyubiquitin chains. The functional outcome of the chain is dependent on the lysine residue involved in the linkage between ubiquitin. The K11- and K48-linked polyubiquitin chains generally lead to proteasomal degradation, while the K63-linked polyubiquitin chain results in the stabilization of protein-protein interaction in most cases [8]. Moreover, some TRIM proteins can mediate SUMOylation, which consists in the attachment of small ubiquitin-related modifier (SUMO) on a lysine [9]. Despite their structural similarities, TRIM proteins exhibit antiviral properties against viruses with diverse infection mechanisms.

The objective of this review is to provide insights into the two complementary modes of regulation by TRIM proteins: their indirect antiviral actions, mediated through various cellular pathways, and their direct effects on the viral life cycle. In a last part, viral strategies emerging from the co-evolution between viruses and TRIMs will be examined.

### 1. Indirect antiviral actions of TRIM proteins

#### 1.1. Increase of antiviral immune signaling by TRIM proteins.

**Fig 2:**
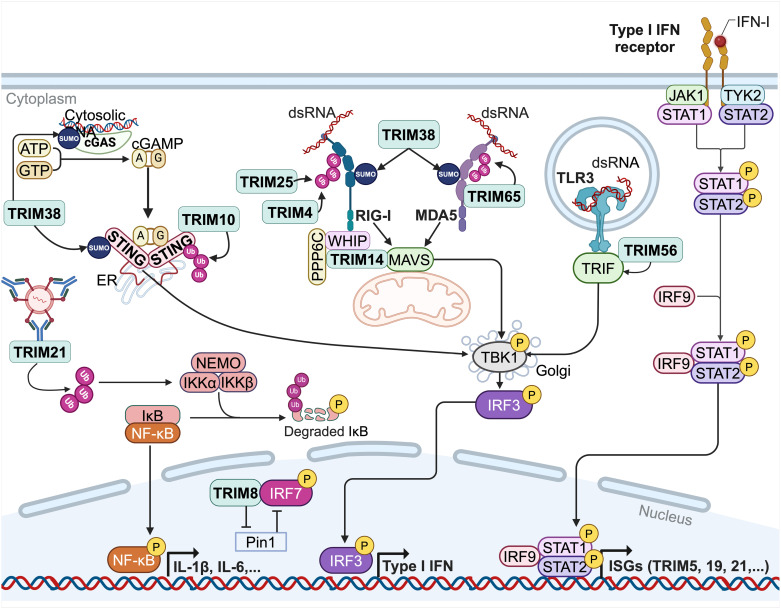
TRIM proteins positively regulate innate immune signaling pathways. Upon recognition of viral DNAs or RNAs, pattern-recognition receptors (PRRs) such as TLR3, cGas/STING and RLRs (RIG-I and MDA5) are activated by TRIM proteins [12,15–20,26,28]. Except for TLR3, this positive regulation is dependent on polyubiquitination or SUMOylation catalyzed by TRIM proteins. TRIM proteins actions on immune signaling pathways result in the promotion of type I interferon production, which in turn activates the expression of many TRIM proteins, through the JAK/STAT signaling pathway. TRIM21 itself detects intracellular opsonized viruses and initiates the NF-κB pathway [25,26]. Created in BioRender. Lucifora, J. (2025) https://BioRender.com/d11y722.

Innate immunity relies on the detection of conserved motif shared by large classes of pathogens, known as pathogen-associated molecular patterns (PAMPs). These PAMPs, depending on their nature (e.g., viral DNA or RNA, structural proteins) are detected by various pattern-recognition receptors (PRRs) such as Toll-like receptors (TLRs) or RIG-I like receptors (RLRs) (Fig 2) [10]. This recognition leads to the recruitment of adaptor proteins, signal transduction and ultimately, activation of genes involved in immune defense.

*1.1.a TRIMs contribute to the activation of diverse PRRs upon viral infection*

TLRs are localized at the cell surface or within endosome and detect viral RNA, DNA or viral protein, leading to the recruitment of the TIR domain-containing adapter inducing IFN-β (TRIF) protein [11]. TRIF mediates a signaling cascade that results in the phosphorylation of Interferon regulatory factor 3 (IRF3) and its subsequent translocation in the nucleus. IRF3 activates the production of type I IFN and the expression of Interferon-stimulated genes (ISGs), which mediate diverse cellular mechanisms to inhibit viral replication. Recent studies have highlighted the critical role of TRIM56 in positively regulating the TLR3 signaling pathway during Hepatitis C Virus (HCV) infection, through its interaction with TRIF [12]. Interestingly, RING-deleted TRIM56 still associates with TRIF and promotes TLR3 signaling, suggesting that the E3 ubiquitin ligase activity is not required [12].

Similarly, viral RNAs are recognized by RLRs (RIG-I and MDA5), which oligomerize and translocate to the mitochondria where they activate the adaptor mitochondrial antiviral signaling (MAVS) proteins [8]. The RLR-MAVS complex promotes the phosphorylation of IRF3 and nuclear factor-kappa B (NF-κB). TRIM proteins modulate RLRs signaling at different stages [2]. Upon double-stranded RNA (dsRNA) detection, TRIM65 ubiquitinates MDA5, promoting the formation of MDA5 aggregates required for its activation [13]. Numerous TRIM proteins, such as TRIM25 [14,15] and TRIM4 [16], are also essential for RIG-I activation, both adding K63-linked polyubiquitin chains to RIG-I. This modification is critical for RIG-I activation and subsequent type I IFN production. The redundant functions of TRIM25 and TRIM4 in RIG-I signaling ensure robustness of this essential immune signaling pathway. In addition to ubiquitination, RIG-I and MDA5 are regulated by TRIM38-mediated SUMOylation [9]. In early infected cells, TRIM38-mediated SUMOylation enhances RIG-I and MDA5 activation, while deSUMOylation at later stages promotes their degradation [17]. Moreover, the interaction between RIG-I and MAVS, essential for the downstream signaling, is mediated by a multi-protein complex comprising TRIM14, WHIP, and PPP6C [18]. TRIM14 binds directly to MAVS, while WHIP interacts with RIG-I, establishing a molecular bridge between RIG-I and MAVS.

In addition to RNA sensors, TRIM proteins are also involved in DNA sensing pathways. Cyclic GMP-AMP synthase (cGAS) is a cytosolic DNA sensor that produces cGAMP as a second messenger upon activation [10]. The detection of cGAMP by the adaptor protein Stimulator of Interferon Genes (STING) promotes its dimerization and translocation to the Golgi apparatus to activate TANK-binding kinase 1 (TBK1). TBK1 phosphorylates IRF3, thereby initiating the production of type I IFN [2]. The regulation of cGas and STING by TRIM38-mediated SUMOylation is similar to that previously described with RIG-I and MDA5 [19]. Additionally, TRIM proteins facilitate immune signaling by promoting protein trafficking between cellular compartments. Kong *et al.* identified a pivotal role for TRIM10 in STING trafficking [20]. Indeed, TRIM10 mediates STING K27- and K29- linked polyubiquitination, which foster STING translocation and aggregation at the Golgi, promoting TBK1 activation. Survival rate of TRIM10 deficient mice was lower than that of control mice following Herpes simplex virus 1 (HSV-1) infection, underscoring the essential role of TRIM10 *in vivo*. Overall, it appears that TRIM proteins can activate PRRs or their adaptor proteins, primarily through polyubiquitination, though not exclusively. Their effectiveness is enhanced by redundancy, as multiple TRIM proteins can have similar effects on a given PRR. Additionally, individual TRIM proteins can engage in multiple immune signaling pathways, supporting a broad-spectrum antiviral defense.

However, some TRIM proteins negatively regulate PRRs or their downstream effector molecules, limiting the inflammatory response. For example, Zhao *et al*. demonstrated that TRIM40, which is expressed in resting macrophages, targets both RIG-I and MDA5 for proteasomal degradation, reducing the production of type I IFN [21]. In contrast, during RNA virus infection, the expression of TRIM40 is downregulated, allowing for a more efficient antiviral response. Another example of this regulatory role of TRIM proteins is TRIM26 [22], which interferes at the end of the immune signaling pathway by promoting IRF3 degradation in the nucleus [23]. Together, these findings highlight the role of TRIM proteins in maintaining a balance between antiviral response and homeostasis.

*1.1.b TRIMs activate Interferon production and are in turn up-regulated by IFN*

Some TRIM proteins promote IFN production either by initiating signaling pathways themselves or by acting directly at the transcriptional level. Very interestingly, TRIM21 displays a distinct mechanism among the TRIM family and might be considered as a PRR with its own distinct signaling pathway, ultimately leading not only to the production of type I IFN and other pro-inflammatory cytokines but also to the proteasomal degradation of viruses [24]. In both immune and non-immune cells, TRIM21 binds with high affinity to intracellular antibodies (Ab) that opsonize a virus [25], activating both NF-κB and IRF pathways through the production of unanchored K63-linked polyubiquitin chains [26]. The engagement of TRIM21 and its bound immune complex toward the proteasomal degradation pathway depends on sequential ubiquitination and deubiquitination, starting with TRIM21 auto-monoubiquitination, followed by the synthesis of anchored K63-linked polyubiquitin chains followed by the synthesis of an anchored K63-linked polyubiquitin chains [27]. In their study, Fletcher *et al.* propose a model in which the proteasome-associated deubiquitinase Poh1 liberates K63-polyubiquitin chains anchored to TRIM21, although they could not directly prove that the chains released by Poh1 activate NF-κB pathway [27]. In contrast, some TRIM proteins act primarily at the transcriptional level, where they regulate IFN response by competing with negative regulators. It has been demonstrated that TRIM8, mainly expressed in the nucleus of plasmacytoid dendritic cells, plays a crucial role in protecting IRF7, a key transcription factor for type I IFN production [28]. These cells constitutively express IRF7, enabling a rapid and massive production of type I IFN. Through polyubiquitination, TRIM8 protects IRF7 from the degradation mediated by the peptidyl-prolyl isomerase Pin1, thus maintaining robust IFN signaling.

It is noteworthy that numerous TRIM proteins activate the IRF pathway, resulting in the production of type I IFN, which in turn also regulate TRIM proteins expression through the JAK/STAT pathway (Fig 2) [29]. TRIM19, also referred to as PML, was the first TRIM protein to be identified as being up-regulated under IFN treatment [30]. Carthagena and colleagues identified 27 TRIM proteins as ISGs, among which 16 were up-regulated by type I IFN, including TRIM5, TRIM21, TRIM25, TRIM56 and TRIM69. In addition, 8 TRIMs were up-regulated by type II IFN, including TRIM19, TRIM22 and TRIM69 (Table 1) [29]. This positive feedback regulation ensures a robust activation of immune defenses against viral infection. Although type III IFN activate a similar signaling pathway as type I IFN, resulting in a comparable transcriptional response [31], little is known about the effects of type III IFN on TRIM expression. Recent findings in duck embryonic cells showed that TRIM25 expression was upregulated in a dose-dependent manner by type III IFN treatment, and in turn, TRIM25 increased the production of type III IFN, as a positive feed-forward regulatory loop [32].

**Table 1:** TRIM proteins upregulated in response to interferon stimulation. Systematic analysis of TRIM genes whose expression is induced by type I and type II interferons in human primary lymphocytes and monocyte-derived macrophages. Data from [29].

Type I interferon	Type II interferon
TRIM2, 5, 6, 14, 19, 20, 21, 22, 25, 26, 31, 34, 35, 38, 56, 58, 69	TRIM19, 20, 21, 22, 25, 26, 56, 69

### 1.2. Viral infection impairment through cytoskeleton reorganization by TRIM proteins

Despite the established role of the cytoskeleton in viral trafficking, particularly through microtubules (MT), the mechanisms by which cells impede this process remain poorly understood. Emerging studies suggest that TRIM proteins may hinder cytoskeletal dynamics, interfering with viral transport [33]. In macrophages, TRIM69 has been recently identified as a restriction factor against a wide range of retroviruses and RNA viruses, namely human immunodeficiency virus-1 (HIV-1), vesicular stomatitis virus (VSV) and SARS-CoV-2 [34]. TRIM69 directly binds to MT, thereby increasing their stability. Moreover, this binding might interfere with dynein transport on stable MT, which is crucial for virus trafficking. TRIM69 action on MT impairs early stages of the viral cycle, though the specific steps affected vary depending on the virus. While both VSV entry and post-entry steps are affected by TRIM69, SARS-CoV-2 and HIV-1 are only impacted at post-entry steps. TRIM69 seems to be the first identified TRIM protein displaying the property to remodel MT to limit viral dissemination. Another study revealed that TRIM11 accelerates HIV-1 uncoating, decreasing reverse-transcription in a MT-dependent manner. However, the correspondent mechanism is still unknown [35]. Other TRIM proteins, such as TRIM18 and TRIM46, interact with MT, yet a potential link with viral infection has not been established [33].

In addition to MT, some viruses rely on intermediate filaments (IF) for their replication. During HSV-1 infection, TRIM43 ubiquitinates the centrosomal protein Pericentrin, resulting in its proteasomal degradation [36]. As the centrosome is associated with the nuclear envelope, a loss of centrosome integrity results in an alteration of the nuclear lamina, which is a type of IF. This alteration induces the repression of viral chromatin expression, which in turn suppresses the reactivation of latent virus or lytic replication. Interestingly, HSV-1 infection up-regulated TRIM43 level, independently of type I IFN stimulation. *TRIM43* gene expression was found to be controlled by the transcription factor Double homeobox 4 (DUX4), whose expression may be reactivated by the integration of HSV-1 into the cell genome [36].

The first section highlights that TRIM proteins indirectly counteract viral infections through diverse mechanisms, including enhancing immune signaling, initiating responses upon detecting opsonized viruses, competing with transcription inhibitors of pro-inflammatory cytokines, and disrupting the viral cycle via cytoskeletal interactions.

## 2. Direct antiviral actions of TRIM proteins

In addition to their influence on immune signaling pathways and cytoskeleton dynamics, TRIM proteins directly restrict viral infection, frequently by promoting the degradation of viral proteins via their shared E3 ubiquitin ligase activity.

### 2.1. TRIM proteins directly interfere with viral cycle

**Fig 3:**
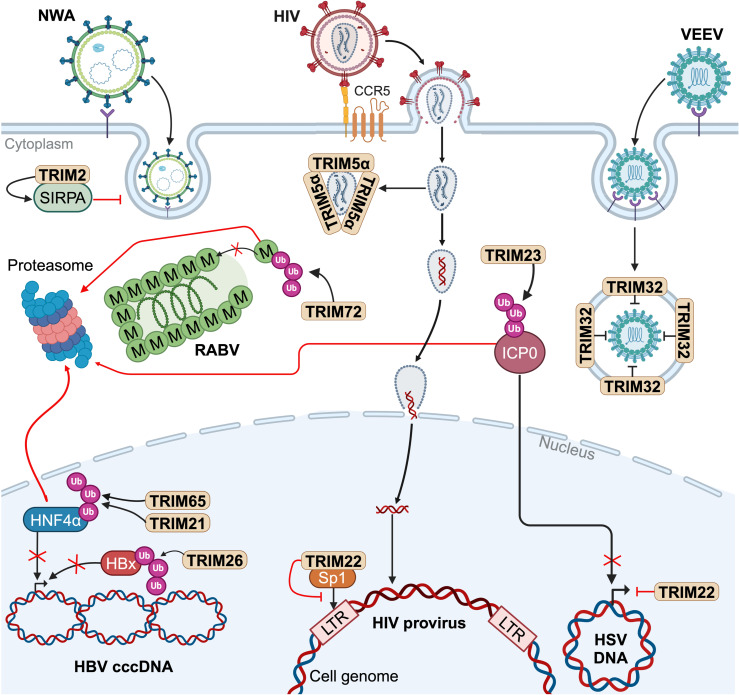
TRIM proteins directly antagonize viruses. In general, TRIM proteins ubiquitinate viral proteins, leading to their proteasomal degradation. TRIM2 inhibits New World Arenavirus (NWA) entry [37]. TRIM32 impairs the uncoating of Venezuelan equine encephalitis virus (VEEV) [38]. TRIM5α forms a shield around HIV capsid [39] and HIV expression is impaired by TRIM22 [48]. Other TRIM proteins degrade transcription factors, such as TRIM21 [40], TRIM26 [44] and TRIM65 [41] which limit the expression of Hepatitis B virus (HBV) genome. TRIM23 [45] and TRIM22 [49] inhibit the expression of Herpes simplex virus (HSV), respectively by promoting the degradation of ICP0 and chromatin compaction. TRIM72 targets the Matrix (M) protein of Rabies virus (RABV) for degradation, preventing virus assembly [47]. Created in BioRender. Lucifora, J. (2025) https://BioRender.com/q00o031.

TRIM proteins can interfere with the viral cycle at various steps, from the entry, replication to viral egress (Fig 3). TRIM proteins impede viral entry at different stages from the endocytosis to the uncoating. For example, TRIM2, which is highly expressed in the brain, impairs New World arenavirus (NWA) entry by interacting with SIRPA, an inhibitor of phagocytosis [37]. Interestingly, the RING domain is not required for this antiviral effect in contrast to the FIL domain, implying that this restriction relies on protein-protein interactions [37]. In contrast, TRIM32 acts at a later step in the entry of Venezuelan equine encephalitis virus (VEEV), an RNA virus [38]. TRIM32, localized at the endosome membrane, leads to an impaired translation of encapsidated RNA, without affecting virus binding or internalization [38]. One proposed hypothesis is that TRIM32 might disturb VEEV uncoating [38]. This strategy of interfering with uncoating, was also described for TRIM5α, which is among the most extensively studied TRIM proteins. The PRY-SPRY domain of rhesus TRIM5α directly binds to HIV-1 capsid, forming a shield around the capsid to prevent the integration of the viral DNA into the genome, independently from E3 ligase activity [39].

Some TRIM proteins interfere with viral transcription through the degradation of viral proteins or cellular transcription factors hijacked by viruses. For example, numerous TRIM proteins impair Hepatitis B virus (HBV) expression and replication. TRIM21 [40] and TRIM65 [41] inhibit HBV replication by targeting for degradation the hepatocyte nuclear factor 4 alpha (HNF4α), a key transcription factor for HBV RNAs synthesis [42]. In addition, the HBx protein of HBV, which is also required for viral transcription [43], is polyubiquitinated by TRIM26, leading to its degradation [44]. However, the role of TRIM65 and TRIM26 as restriction factors for HBV replication has been demonstrated only in hepatocyte cell lines, including Huh7 and HepG2 cells, which are not completely representative of mature hepatocytes. Another study pointed the involvement of TRIM23 in the degradation of Infected Cell Polypeptide 0 (ICP0) [45], an HSV protein that promotes early viral transcription [46]. TRIM23 targets ICP0 for proteasomal degradation through K11- and K48-linked polyubiquitin chains, limiting HSV expression and replication [45].

Finally, viral assembly and release can also be impaired by various TRIM proteins. A recent article describes TRIM72 as an important restriction factor of rabies virus (RABV) in neurons [47]. The SPRY domain of TRIM72 directly interacts with the Matrix protein of RABV, while the RING domain catalyzes its polyubiquitination, resulting in the proteasomal degradation of the Matrix protein and, ultimately, the disruption of RABV assembly [47].

Some TRIM proteins exhibit versatility by targeting multiple proteins, impeding the replication of diverse viruses. A prime example of this multifunctionality is TRIM22, which restricts HIV-1 infection by preventing the transcription factor Sp1 from binding to the LTR promoter, thereby inhibiting viral gene expression [48]. In addition, TRIM22 suppresses HSV-1 expression by promoting chromatin compaction [49]. These observations once more illustrate the robustness and adaptability of TRIMs defense mechanism.

### 2.2. TRIMs act as selective autophagy receptors

**Fig 4:**
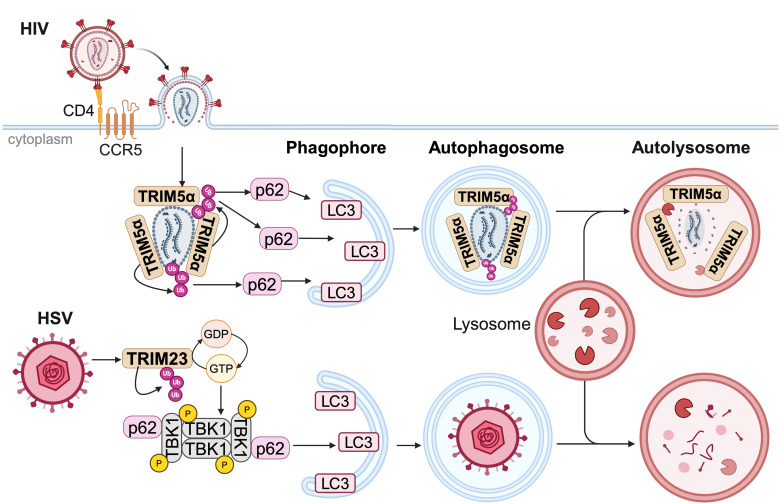
TRIM proteins initiate the autophagic degradation of certain viruses. TRIM5α binds to HIV capsid and catalyzes the formation of polyubiquitin chains leading to the recruitment of p62 and LC3, both of which are involved in autophagosome formation [39]. The Herpes simplex virus (HSV) infection triggers TRIM23 auto-ubiquitination which activates its GTPase activity [54]. The GTP-GDP cycle promotes TBK1 auto-phosphorylation, which in turn leads to the association with p62. The autophagosome then fuses with a lysosome and the combined action of lysosomal enzymes and low pH degrades viral components. Created in BioRender. Lucifora, J. (2025) https://BioRender.com/r82i202.

Viral infection can trigger autophagy, which results in the degradation of viral components within autophagosomes that fuse with lysosomes. Additionally, autophagy promotes antigen presentation and the production of pro-inflammatory cytokines [50]. Several TRIM proteins have been demonstrated to participate in the autophagy process during viral infection and may be considered as receptors for selective autophagy (Fig 4) [6].

We mentioned above rhesus TRIM5α, which can shield the HIV capsid without polyubiquitination. Conversely, when TRIM5α catalyzes K-63-linked polyubiquitination, this modification recruits the autophagic adaptor protein p62, facilitating interaction with the autophagosome marker LC3 [39]. This autophagic process leads to capsid disassembly, inhibiting reverse transcription of HIV. Nevertheless, while it is now well accepted that rhesus TRIM5α strongly restricts HIV infection, the role of human TRIM5α has been debated [51] since human TRIM5α expressed in HeLa cells had very little inhibitory effect on HIV infection [52]. However, Ribeiro *et al*. recently showed that human TRIM5α could target HIV-1 for autophagic degradation in a specific subset of dendritic cells, the Langerhans cells [53]. This restriction was dependent on the uptake of HIV-1 through the Langerin receptor, which is part of the C-type-lectin-receptor family and is specific to this cell type. A model currently emerging proposes rhesus TRIM5α as a general restriction factor while human TRIM5α would be cell-specific [51]. TRIM23 promotes the activation of a key cellular autophagy receptor, p62, through a distinct mechanism, which relies on GTPase activity. TRIM23 has been identified as a restriction factor of DNA viruses, including HSV-1, as well as RNA viruses such as Influenza A virus (IAV) and EMCV [54]. TRIM23 displays both E3 ubiquitin ligase activity via the RING domain and GTPase activity via the ARF domain, which is a unique characteristic amongst TRIM members. The RING domain catalyzes the K27-linked polyubiquitination of the ARF domain, activating the GTPase activity. GTP hydrolysis promotes the interaction with p62 and TBK1, both proteins playing a role in autophagy initiation [50]. Moreover, the conformational changes induced by the GTP hydrolysis may induce the multimerization of TBK1, which in turn facilitates its autophosphorylation and subsequent activation. Other TRIM proteins, such as TRIM16 [55], also regulate autophagy pathway but their action in viral infection has not been demonstrated yet.

Altogether, TRIM proteins appear to play a central role in different cellular degradation pathways, orchestrating the selective clearance of viral components through precise mechanisms such as polyubiquitination.

## 3. An arms race between viruses and TRIM proteins

**Fig 5:**
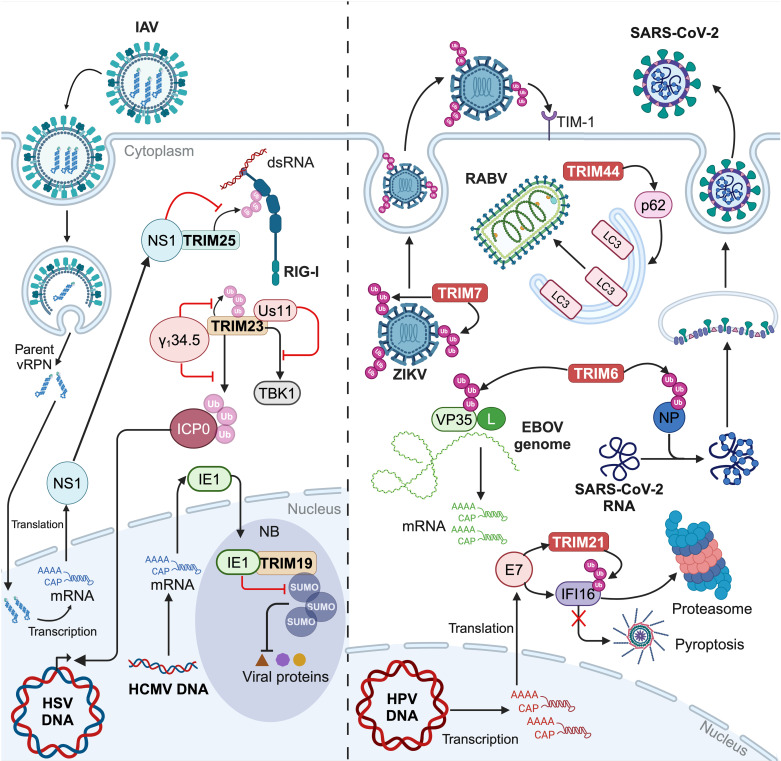
Examples of viruses escaping the restriction mediated by TRIM proteins. Left side: Viruses inhibit the antiviral action of TRIMs. NS1 protein of Influenza A virus (IAV) impairs the activation of RIG-I by TRIM25 [56]. TRIM23 is antagonized by HSV proteins γ_1_34.5 [45] and Us11 [63], removing the restriction on HSV expression and blocking autophagy initiation. IE1 protein of human cytomegalovirus (HMCV) impedes the SUMOylation of TRIM19, leading to nuclear body (NB) disruption and release of viral proteins [58]. Right side: Viruses take advantages of TRIM-mediated modifications. Polyubiquitination of the Zika virus (ZIKV) envelope protein promotes its interaction with the TIM-1 receptor [64]. TRIM6-mediated modification of VP35 stabilizes L polymerase on the Ebola virus (EBOV) genome [65]. In addition, TRIM6 facilitates the association of the nucleoprotein (NP) of SARS-CoV-2 with the viral genome [67]. TRIM44 promotes the initiation of autophagy, which facilitates RABV replication [68]. Human papillomavirus (HPV) E7 protein enables polyubiquitination of IFI16 by TRIM21, inhibiting inflammasome assembly and pyroptosis [70]. Created in BioRender. Lucifora, J. (2025) https://BioRender.com/q85j502.

Co-evolution between viruses and their hosts has resulted in the emergence of viral mechanisms that enable them to evade restriction factors. Such strategies can either inhibit the antiviral activities of TRIM proteins or even exploit TRIMs to promote infection (Fig 5).

### 3.1. Viruses interfere with TRIM proteins, inhibiting their antiviral activities

Several viruses share the same strategy, consisting in a direct binding to the coiled-coil domain of TRIM proteins, preventing their interaction with a substrate. As mentioned in the first section, TRIM25 is a crucial regulator of RIG-I [15]. Gack *et al.* demonstrated that TRIM25 was inhibited by the Non-structural protein 1 (NS1) of IAV [56]. NS1 forms a complex with TRIM25, by directly binding to its coiled-coil domain. This interaction impedes the appropriate positioning of the PRY-SPRY domain and the RING domain, required to mediate the ubiquitin transfer [57]. However, TRIM25 is still capable of producing unanchored K63 polyubiquitin chains, but they are unable to activate RIG-I. Similarly, during human cytomegalovirus (HCMV) infection, the viral immediate early protein 1 (IE1) binds to the coiled-coil domain of TRIM19/PML [58]. TRIM19 normally forms nuclear bodies which sequester viral proteins [59]. However, the interaction between TRIM19 and IE1 impairs the polySUMOylation of TRIM19, which is required for the formation and maintenance of the nuclear bodies. IE1 might prevent polySUMOylation by steric hindrance, blocking the access to the polySUMOylation site on TRIM19. These evasion strategies are not limited to preventing TRIM proteins from accessing their substrates, as some viruses induce TRIM degradation. Bharaj *et al.* showed that the Matrix protein of Nipah virus interacts with the SPRY domain of TRIM6 and reduces its protein levels, limiting the activation of the kinase IKKε, and consequently impairing the phosphorylation of IRF3 and type I IFN production [60]. Although the molecular mechanism underlying TRIM6 degradation remains unclear, experiments using specific inhibitors suggest that this process is independent of both proteasomal and lysosomal pathways [60].

TRIM22 has been shown to inhibit the activity of the HBV core promoter [61] and HBV infection was shown to counteract TRIM22 through another type of strategy. Indeed, instead of directly interfering with the protein activity, HBV interfere with the expression of TRIM22 at the transcriptional level [62]. It was demonstrated that the expression of the viral protein HBx resulted in methylation in the 5' UTR region of *TRIM22* gene [62]. This epigenetic modification reduces the binding affinity of the 5' UTR region for interferon regulatory factor 1 (IRF1), impairing *TRIM22* transcription in response to IFNs stimulation.

Some escape mechanisms are highly robust, as a single virulence factor can impede two distinct antiviral effects of a given TRIM protein. As an example, HSV-1 has evolved to antagonize TRIM23 restriction, which has been previously described, by interfering with the degradation of the viral protein ICP0 [45]. A second viral protein γ_1_34.5, directly binds ICP0, preventing it from undergoing polyubiquitination and proteasomal degradation. γ_1_34.5 also binds the ARF domain of TRIM23, inhibiting its autoubiquitination, required to initiate autophagy. In addition, the HSV-1 Us11 protein also targets TRIM23-mediated autophagy [63]. Similar to γ_1_34.5, Us11 binds to the TRIM23 ARF domain, preventing the interaction with TBK1, and consequently inhibiting the formation of autophagosomes. It appears that, as a given TRIM protein can target multiple viral proteins and conversely, some TRIM proteins exert a redundant effect on the same viral protein, the same conclusion can be drawn regarding the action of viral proteins. Indeed, a given viral protein can interfere with diverse TRIM proteins and different viral proteins target the same TRIM protein, conferring on certain viruses a robust capacity to impede both direct and indirect TRIM actions.

### 3.2. Some modifications of viral proteins by TRIM proteins can favor viral replication

Surprisingly, instead of blocking TRIM activity, some viruses exploit the modifications conferred by TRIM proteins either to directly facilitate certain steps of the viral cycle or to redirect cellular pathways for their benefit, including autophagy and inflammatory pathways.

K63-linked polyubiquitination catalyzed by TRIM7 on the Envelope (E) of Zika virus (ZIKV) enhances viral entry into human cells [64]. The study by Giraldo *et al.* showed that ZIKV infection induces a reorganization of intracellular membranes accompanied by a relocalization of TRIM7 to these membranes, where it can interact with the E protein. The polyubiquitination of this viral protein promotes its binding to the receptor TIM-1, thus improving viral entry. Interestingly, ubiquitination of the E protein had no impact on infection in *Aedes aegypti* mosquitoes, suggesting that this modification may be involved in determining species tropism.

A given TRIM protein can be hijacked by disparate viruses to promote different steps of their life cycle, underscoring the convergence of robust viral escaping strategies. The polymerase cofactor VP35 of Ebola virus (EBOV) is ubiquitinated by TRIM6, promoting viral transcriptase activity [65]. This modification might induce the interaction of VP35 with the large polymerase L, ensuring polymerase stability [66]. Similarly, SARS-CoV-2 hijacks TRIM6 to ubiquitinate its Nucleocapsid Protein (NP), promoting its binding to viral RNA and facilitating assembly [67].

Autophagy acts as a double-edged sword, with the potential to either impair or promote viral infection, making this pathway susceptible to viral hijacking [50]. A recent study demonstrated that the silencing of TRIM44 inhibited RABV replication, while its overexpression induced higher virus titers [68]. TRIM44 facilitates RABV replication by activating the autophagy pathway. TRIM44 may promote oligomerization of the autophagy receptor p62, triggering the recruitment of LC3 [69]. Furthermore, additional research is required to elucidate the mechanisms by which autophagy facilitates RABV replication.

Given that TRIM proteins regulate a variety of immune signaling pathways, viruses can interfere with these pathways as well. The oncoprotein E7 of Human papillomavirus (HPV) recruits both TRIM21 and interferon-gamma inducible protein 16 (IFI16) which is known to mediate inflammasome assembly and initiate pyroptosis, an inflammatory cell death that occurs upon infection [70]. TRIM21 targets IFI16 for degradation through polyubiquitination, resulting in the escaping of cell pyroptosis for HPV. In addition to inflammasome-related pathways, viruses also commonly target the type I IFN signaling cascade. Laurent-Rolle *et al.* identified a role for TRIM23 in mediating this IFN antagonism during yellow fever virus (YFV) infection [71]. Indeed, TRIM23 adds K63-polyubiquitin chains to the viral nonstructural protein 5 (NS5), allowing its interaction with STAT2. This interaction, which occurs only under type I IFN stimulation, impairs the engagement of the complex STAT1-STAT2-IRF9 in the activation of ISGs expression (Fig 2), inhibiting the final step of type I IFN signaling [71]. Viruses can also harness some TRIM proteins that act as negative regulators of immune signaling pathways. For example, TRIM29, which is expressed in alveolar macrophages [72], has been shown to target the adaptor protein STING for proteasomal degradation [73]. This process helps to control inflammation, which is crucial in cells at the interface with the external environment, thus exposed to inflammatory particles and pathogens. However, Xing *et al.* showed that EBV infection and vaccinia virus DNA stimulate TRIM29 expression in human airway epithelial cells, leading to in an impaired immune response due to STING degradation [74].

To summarize, it appears that, as a reflection of the TRIM proteins action both directly on viral proteins and indirectly by modulating cellular pathways, viruses escaping strategies hijack TRIM proteins either to directly promote their viral life cycle or to redirect cellular pathways. Such proviral effects of TRIM proteins highlight their potential as therapeutic targets for antiviral interventions and immune regulation.

## 4. Conclusion

Although TRIM proteins share a conserved structure, they restrict a wide range of viral infections through diverse and complementary mechanisms. This review underscores the multifaceted roles of TRIMs, which range from modulating immune signaling pathways and reorganizing the cytoskeleton to directly degrading viral components through proteasomal or autophagic pathways. The breadth of these antiviral strategies, coupled with their capacity for redundancy and versatility, highlights TRIM proteins as a robust and adaptable line of defense, but also highly susceptible to viral hijacking. TRIM proteins are frequent targets of viral evasion strategies, with many viruses developing sophisticated molecular mechanisms to subvert or even exploit TRIM proteins functions. However, as the majority of studies rely only on cellular models, which are not fully representative of the complexity of TRIM protein-mediated defense during a viral infection, further investigations using *in vivo* models are needed to strengthen current observations. Moreover, the roles of TRIM proteins have been identified using a combination of overexpression systems and loss-of function approaches, both presenting some limitations. The druggability of TRIM proteins to treat viral infections presents a significant challenge, as emerging evidence indicate their role in cancer development and resistance to treatments [75]. For instance, TRIM32, identified as a restriction factor against VEEV [38], is linked to poor survival in gastric cancer, possibly through its role in AKT phosphorylation and enhanced glucose transport in cancer cells [76]. Similarly, TRIM23, associated with poor prognosis in colorectal cancer, promotes tumor progression by degrading the pivotal tumor suppressor p53 [77]. Understanding these dual roles in host-pathogen interaction and oncology is crucial to safely targeting TRIMs for therapeutic purposes.

## Abbreviations

ARF: ADP-ribosylation factor
DUX4: double homeobox 4
EBOV: Ebola virus
HBV: hepatitis B virus
HCV: hepatitis C virus
HIV: human immunodeficiency virus
HPV: human papillomavirus
HSV: herpes simplex virus
IAV: influenza A virus
ICP0: infected cell polypeptide 0
IFN: interferon
ISGs: interferon-stimulated genes
MAVS: mitochondrial antiviral signaling
MT: microtubules
NP: nucleocapsid protein
PAMPs: pathogen-associated molecular patterns
PRRs: pattern-recognition receptors
RABV: rabies virus
RLRs: RIG-I like receptors
STING: stimulator of interferon genes
SUMO: small ubiquitin-related modifier
TLRs: toll-like receptors
TRIM: tripartite motif
VEEV: Venezuelan equine encephalitis virus
VSV: vesicular stomatitis virus
YFV: yellow fever virus
ZIKV: Zika virus
